# Novel Functional Food Properties of Forest Onion (*Eleutherine bulbosa* Merr.) Phytochemicals for Treating Metabolic Syndrome: New Insights from a Combined Computational and In Vitro Approach

**DOI:** 10.3390/nu16101441

**Published:** 2024-05-10

**Authors:** Happy Kurnia Permatasari, Nuril Farid Abshori, Rony Abdi Syahputra, Urip Harahap, Nurlinah Amalia, Dian Aruni Kumalawati, Nelly Mayulu, Nurpudji Astuti Taslim, Trina Ekawati Tallei, Raymond Rubianto Tjandrawinata, Elvan Wiyarta, Adriyan Pramono, Bonglee Kim, Apollinaire Tsopmo, Lluis Serra-Majem, Fahrul Nurkolis

**Affiliations:** 1 Department of Biochemistry and Biomolecular, Faculty of Medicine, University of Brawijaya, Malang 65145, Indonesia; 2 Medical Study Program, Faculty of Medicine, State Islamic University of Maulana Malik Ibrahim (UIN Maulana Malik Ibrahim), Malang 65144, Indonesia; 3 Department of Pharmacology, Faculty of Pharmacy, Universitas Sumatera Utara, Medan 20155, Indonesia; 4 Master Program of Biomedical Science, Faculty of Medicine, Brawijaya University, Malang 65151, Indonesia; 5 Department of Biological Sciences, Faculty of Sciences and Technology, State Islamic University of Sunan Kalijaga (UIN Sunan Kalijaga), Yogyakarta 55281, Indonesiafahrul.nurkolis.mail@gmail.com (F.N.); 6 Department of Nutrition, Faculty of Health Science, Muhammadiyah Manado University, Manado 95249, Indonesia; 7 Division of Clinical Nutrition, Department of Nutrition, Faculty of Medicine, Hasanuddin University, Makassar 90245, Indonesia; 8 Department of Biology, Faculty of Mathematics and Natural Sciences, Sam Ratulangi University, Manado 95115, Indonesia; 9 Department of Biotechnology, Faculty of Biotechnology, Atma Jaya Catholic University of Indonesia, Jakarta 12930, Indonesia; 10 Department of Neurology, Faculty of Medicine, Universitas Indonesia-Dr. Cipto Mangunkusumo National Hospital, Jakarta 10430, Indonesia; 11 Department of Nutrition, Faculty of Medicine, Diponegoro University, Semarang 50275, Indonesia; 12 Center of Nutrition Research (CENURE), Diponegoro University, Semarang 50275, Indonesia; 13 Department of Pathology, College of Korean Medicine, Kyung Hee University, Seoul 02447, Republic of Korea; 14 Food Science Program, Department of Chemistry, Institute of Biochemistry, Carleton University, 1125 Colonel by Drive, Ottawa, ON K1S 5B6, Canada; 15 Centro de Investigación Biomédica en Red Fisiopatología de la Obesidad y la Nutrición (CIBEROBN), Institute of Health Carlos III, 28029 Madrid, Spain; 16 Research Institute of Biomedical and Health Sciences (IUIBS), University of Las Palmas de Gran Canaria, 35001 Las Palmas, Spain

**Keywords:** forest onion, metabolic syndrome, diabetes, obesity, network pharmacology, phytochemicals, functional food, preadipocyte 3T3-L1, mouse cell line

## Abstract

Metabolic syndrome is a global health problem. The use of functional foods as dietary components has been increasing. One food of interest is forest onion extract (FOE). This study aimed to investigate the effect of FOE on lipid and glucose metabolism in silico and in vitro using the 3T3-L1 mouse cell line. This was a comprehensive study that used a multi-modal computational network pharmacology analysis and molecular docking in silico and 3T3-L1 mouse cells in vitro. The phytochemical components of FOE were analyzed using untargeted ultra-performance liquid chromatography–tandem mass spectrometry (UPLC-MS). Next, an in silico analysis was performed to determine FOE’s bioactive compounds, and a toxicity analysis, protein target identification, network pharmacology, and molecular docking were carried out. FOE’s effect on pancreatic lipase, α-glucosidase, and α-amylase inhibition was determined. Finally, we determined its effect on lipid accumulation and MAPK8, PPARG, HMGCR, CPT-1, and GLP1 expression in the preadipocyte 3T3-L1 mouse cell line. We showed that the potential metabolites targeted glucose and lipid metabolism in silico and that FOE inhibited pancreatic lipase levels, α-glucosidase, and α-amylase in vitro. Furthermore, FOE significantly (*p* < 0.05) inhibits targeted protein expressions of MAPK8, PPARG, HMGCR, CPT-1, and GLP-1 in vitro in 3T3-L1 mouse cells in a dose-dependent manner. FOE contains several metabolites that reduce pancreatic lipase levels, α-glucosidase, α-amylase, and targeted proteins associated with lipid and glucose metabolism in vitro.

## 1. Introduction

Non-communicable diseases (NCDs) have emerged as the primary causes of morbidity and mortality in both industrialized and developing nations. Of all NCDs, metabolic syndrome is a true worldwide plague. Metabolic syndrome comprises a collection of symptoms including obesity, dyslipidemia, hyperglycemia, and hypertension, making it the most significant challenge to public health. The global prevalence of metabolic syndrome is 31%, and it is associated with a 2-fold increased risk of developing coronary heart disease and a 1.5-fold increased risk of death [1]. The estimated prevalence of metabolic syndrome in adults in 2017 was 20–25% globally [2]. Indeed, the magnitude of an individual’s risk of developing metabolic syndrome can be estimated using the Framingham score, which is primarily based on levels of high-density lipoprotein (HDL) cholesterol, blood pressure, and diabetes mellitus [3]. According to WHO criteria, a diagnosis of metabolic syndrome requires the presence of two additional risk factors, such as obesity, hyperglycemia, hypertension, high serum triglycerides, decreased serum high-density lipoprotein (HDL) cholesterol, or microalbuminuria, in addition to signs of insulin resistance [4].

The incidence of metabolic syndrome runs parallel to the incidences of type 2 diabetes mellitus and obesity. Based on CDC data from 2017, 30.2 million adults (individuals aged over 18 years) in the USA were diagnosed with type 2 diabetes mellitus. The International Diabetes Federation Diabetes Atlas shows that the worldwide prevalence of diabetes was 8.8% in 2015 and is expected to continue to increase to 10.4% in 2040. The North American and Caribbean region is recorded as having the highest number of diabetes sufferers, containing more than half of all people diagnosed with diabetes worldwide [1]. In line with the high incidence of diabetes, obesity rates follow a similar pattern. A 2015 global survey showed that 73 out of 195 countries in the world have recorded cases of obesity, which affects approximately 604 million adults and 108 million children. This poll indicates that obesity is no longer solely associated with wealth. In nations with a low socioeconomic index (SDI), the prevalence of obesity in young males (ages 25–29) has increased at the highest rate [5]. This result can be attributed to a combination of changes in diet, a sedentary lifestyle, the food environment, and social–cultural influences [6]. To address this issue concretely, multifaceted strategies, such as promoting healthy eating, encouraging physical activity, regulating the food environment, healthcare intervention, community engagement, and policy advocacy, are needed at the individual, community, and policy levels. Metabolic syndrome is three times more likely to develop with diabetes and obesity than with diabetes alone. Globally, the estimated prevalence of metabolic syndrome is projected to comprise 25% of the world’s population [7].

The main therapy for treating metabolic syndrome is still inadequate due to the complexity of the condition [8]. Existing treatments focus on addressing individual components of metabolic syndrome, such as reducing blood glucose levels, managing hyperglycemia, controlling blood pressure, or promoting weight loss [9]. Researchers are continuing their efforts to develop potential therapies that can address all these conditions simultaneously [10]. Therefore, the widening gap between the incidence of metabolic syndrome and existing therapy contributes increasingly to the annual increase in the prevalence of metabolic syndrome, particularly in cases involving diabetes and obesity [11]. The primary challenge for researchers is to design a therapy that can comprehensively address the factors causing metabolic syndrome while minimizing side effects and drug interactions. Unfortunately, pharmacologically focused therapies have high potential for drug interactions and side effects [12]. An alternative worth considering is harnessing the potential of natural foods and remedies, especially given the abundance of such resources in Indonesia. One such natural ingredient that can be explored is the forest onion *Eleutherine bulbosa* L. [13].

*Eleutherine bulbosa* L. is a plant species belonging to the family Iridaceae. It is native to tropical regions of South America, Asia, and Africa [14]. While it is not typically consumed as food, it has garnered attention for its potential medicinal properties, particularly in traditional medicine practices. Several previous studies identified the efficacy of forest onion as an antioxidant and anticancer agent, leaving open the possibility that forest onion may also possess effects against metabolic syndrome. Da Silva (2024) uncovered the potential of *E. bulbosa* as an antioxidant and anticancer agent due to its bioactive compounds in the form of flavonoids, anthocyanins, and quinones based on in vitro, in vivo, and in silico studies [15]. Another study by Lubis (2017) using a colon cancer cell line in vitro demonstrated that *E. bulbosa* exhibited cytotoxic activity against cancer cells [16]. The antioxidant effect of *E. bulbosa* was studied by Shi (2019) in vitro, revealing that *E. bulbosa* contains bioactive compounds such as flavonoids and phenols as antioxidant agents [17]. On the other hand, Herman et al. (2024) also successfully document the proximate contents of *E. bulbosa* and report its in vivo dose-dependent antidiabetic property [18].

At present, research examining the anti-metabolic syndrome effects of forest onion is still lacking and underdeveloped. Therefore, new research is needed to comprehensively investigate forest onion, particularly its biochemical properties, as a potential agent for combating metabolic syndrome [13]. This study aims to conduct a comprehensive exploration of forest onion, particularly its biochemical properties, to address the knowledge gap concerning its potential as an anti-metabolic syndrome agent in addition to its established efficacy as an antioxidant and anticancer agent. This research ensures a robust and reliable evaluation of the therapeutic potential of forest onion or *E. bulbosa* in managing metabolic syndrome via a comprehensive multi-modal computational network pharmacology analysis, molecular docking, and the in vitro inhibition of lipid and glucose metabolic enzymes, which have never been reported before.

## 2. Materials and Methods

### 2.1. Preparation and Extraction of Forest Onion

Samples of forest onion or dayak onion bulbs (*E. bulbosa* Merr.) were obtained from an online market, and their botanical identification and authentication were carried out at the Biochemistry and Biomolecular Laboratory of the Faculty of Medicine, Universitas Brawijaya, Indonesia, and matched against the National Center for Biotechnology Information (NCBI) Taxonomy ID 1210469 (NCBI: txid1210469) database. The authors state and confirm that the sample collection was approved by local authorities and complies with relevant national and “IUCN Policy Statement on Research Involving Species at Risk of Extinction” guidelines. Each technique used in this study complied with applicable rules and regulations for in vitro and plant research. The *E. bulbosa* samples were washed with distilled water, cleaned, and dried in a Memmert Incubator IN55 oven (Schwabach, Germany) at 50 °C for 3 × 24 h. A sample size reduction was carried out using a blender (CosmosBlender 2 L ReBlendHigh Speed Hand Blender; Tangerang, Indonesia), producing a coarse simplicia powder. Then simplicia powder was extracted via the maceration method. Furthermore, as much as 200 g of *E. bulbosa* simplicia powder was macerated using two liters of a 96% ethanol (ethyl alcohol, C_2_H_5_OH, Sigma-Aldrich (Darmstadt, Germany)) solvent for 3 × 24 h with occasional shaking. The filtrate was then filtered, re-macerated, and evaporated using a rotary evaporator at a temperature of 50 °C to produce a thick extract of *E. bulbosa* or forest onion extract (FOE). The FOE was then stored in aluminum foil for use in follow-up tests. This extraction method was performed in reference to similar studies that have been published [19].

### 2.2. Identification of Metabolite–Peptide Profile via Untargeted Metabolomic Profiling

A metabolomic profile analysis comprising untargeted profiling and compound identification was carried out by laboratory technicians and referred to similar research protocols [20]. An Ultimate 3000LC was used in combination with a Q Exactive MS (Thermo Fisher, Waltham, MA, USA), temp functional centrifugation (Eppendorf, Hamburg, Germany), ACQUITY UPLC HSS T3 (100 × 2.1 mm × 1.8 μm), acetonitrile (Merck, Darmstadt, Germany), methanol (Merck, Darmstadt, Germany), and formic acid (Sigma-Aldrich, Darmstadt, Germany). The samples were liquefied, and 50 mg of each sample was weighed precisely into a tube, supplemented with 800 μL of 80% methanol (methyl alcohol, CH_3_OH) with a vortex for 90 s, and sonicated for 30 min at 4 °C. All samples were then kept at −40 °C for 1 h. After that, each sample was vortexed for 30 s, stored for 30 min, and centrifuged at 12,000 rpm and 4 °C for 15 min. Finally, 200 μL of supernatant was transferred to a vial for LC-MS analysis. Ultra-performance liquid chromatography–tandem mass spectrometry (UPLC-MS) was performed using an Ultimate 3000LC combined with a Q Exactive MS (Thermo Fisher, Waltham, MA, USA) and filtered using electrospray ionization–mass spectrometry (ESI-MS). The LC system consisted of an ACQUITY UPLC HSS T3 (100 × 2.1 mm, 1.8 μm) and the Ultimate 3000LC. The mobile phase consisted of solvent A (0.05% formic acid–water) and solvent B (acetonitrile) with a gradient elution (0–1.0 min, 95% A; 1.0–12.0 min, 95–5% A; 12.0–13.5 min, 5% A; 13.5–13.6 min, 5–95% A; and 13.6–16.0 min, 95% A). The mobile phase flow rate was 0.3 mL/min. The column temperature was maintained at 40 °C, and the sample manager temperature was set at 4 °C. A 40 μL volume of metabolite FOE was injected into the system for each run. The mass spectrometry parameters in positive ion mode (ESI+) and negative ion mode (ESI−) used are presented in the following lists. ESI+: heating temperature, 300 °C; casing gas flow rate, 45 arb; Aux gas flow rate, 15 arb; sweep gas flow rate, 1 arb; spray voltage, 3.0 KV; capillary temperature, 350 °C; and RF S-lens rate, 30%. ESI−: heating temperature, 300 °C, casing gas flow rate, 45 arb; Aux gas flow rate, 15 arb; sweep gas flow rate, 1 arb; spray voltage, 3.2 KV; capillary temperature, 350 °C; and RF S-lens rate, 60%.

### 2.3. In Silico Study Assessment

#### 2.3.1. Prediction of Bioactive Compound Activities, Toxicity Analysis, and Drug-Likeness

Using the WAY2DRUG PASS prediction tool (http://www.pharmaexpert.ru/passonline/predict.php, accessed on 20 February 2024) for metabolic syndrome treatment, the compounds obtained from FOE were examined for potential bioactivity. This method uses an SAR analysis to target the insulin promoter by contrasting input chemicals with compounds that are known to have a certain potency [21]. When the compound’s Pa value is more than 0.4, it is expected to have significant potential, for example, as an antidiabetic agent, due to its similarity to compounds in the database. The Pa value (the probability of being active) represents the output prediction score obtained from the web, which shows the potency of the compound being tested. The Pa value employed in the study was restricted to >0.4 since it represents the accuracy of the prediction function obtained, with a larger Pa value indicating greater accuracy. Additionally, a number of pharmacokinetic factors that are crucial in drug development for evaluating the possible toxicity of a medicine are determined by toxicity and drug-likeness analyses. Each ligand’s drug similarity characteristics were determined using Lipinski’s Rule of Five (Ro5). This analysis was conducted using the SMILES notation of each compound as input for the Protox II database (https://tox-new.charite.de/protox_II/index.php?site=compound_input, accessed on 20 February 2024) and the ADMETLab 2.0 database (https://admetmesh.scbdd.com/service/evaluation/index, accessed on 20 February 2024) [22,23,24]. Supplementary Table S1 displays the SMILES notation for each compound, which was retrieved from PubChem (https://pubchem.ncbi.nlm.nih.gov, accessed on 20 February 2024).

#### 2.3.2. Target Protein Identification and Analysis

The SuperPred target analysis tool (https://prediction.charite.de/, accessed 20 February 2024) was used to perform a target analysis of the FOE. By inserting the SMILES notation for each chemical, the cut-off score for SuperPred Target was established at 80% (ranging from 0% to 100%) for the model’s likelihood and accuracy (Table S1) [25,26]. The Open Targets database, accessible at http://www.opentargets.org/, provided the genes and proteins linked to metabolic syndrome on 20 February 2024. A Venn diagram was then used to map the targets of the FOE and targets connected to the disease in order to determine where the associated targets intersected. The DAVID web server (https://david.ncifcrf.gov/), accessed on 20 February 2024, was used to target-annotate the FOE, with an emphasis on biological processes and Kyoto Encyclopedia of Genes and Genomes (KEGG) pathways [27].

#### 2.3.3. Network Pharmacology Analysis

The STRING (Search Tool for Retrieval of Interacting Genes/Proteins) database was used to analyze relationships between the target proteins derived from the FOE and metabolic syndrome [28]. The target proteins derived from the FOE and protein–metabolism intersections found using the STRING (Search Tool for Retrieval of Genes/Proteins) database were used as inputs. These intersections included the insulin promoter receptor, which is known to be strongly associated with the incidence of metabolic syndrome. Homo sapiens (humans) was chosen as the organism in the STRING Database study, and a high confidence score criterion of 0.9 was used to guarantee strong interactions. After the analysis was completed, data were downloaded from the STRING database in TSV format. CytoScape Version 3.10.1 was then used to process the data for a more in-depth analysis, allowing for the exploration of important network parameters like degree, betweenness centrality, and closeness centrality between receptors [29].

#### 2.3.4. Molecular Docking Simulation

Blind docking using cavity detection as guidance (CB-Dock2, an improved CB-Dock server for protein–ligand blind docking) was used to perform a docking simulation. This technique combines homologous template fitting, docking, and cavity identification. The docking process adhered to the guidelines provided in other publications [30,31]. Using CB-Dock2 for molecular docking, CB-Dock2 is a protein–ligand docking approach that automatically locates binding sites, calculates their center and size, and modifies the docking box size in response to the query ligands. CB-Dock accelerates and improves the accuracy of the docking process by predicting the binding sites of target proteins using a curvature-based cavity detection approach (CurPocket) and the binding poses of query ligands using CB-Dock2 [30,31]. In addition, receptors that are found to have the highest degree of centrality—including those that have been linked to signaling pathways—are utilized for further examinations in molecular docking.

MAPK8 (3ELJ), PPARG (8BF1), HMGCR (2R4F), CPT-1 (1NDB), and GLP-1 (4ZGM) were the enzymes or proteins that were employed. By default, the CB2-Dock Server removed water molecules and other heteroatoms from the uploaded protein structures prior to docking. Ligands were obtained from Pub-Chem in .sdf form (https://pubchem.ncbi.nlm.nih.gov, accessed on 20 February 2024); compounds not found in PubChem were visualized using 22.2.0 ChemDraw MacBook Version. All receptor or target proteins were obtained in .pdb format from the RSCB Protein Data Bank (https://www.rcsb.org; accessed on 20 February 2024).

### 2.4. In Vitro Study Assessments

#### 2.4.1. Antiobesity Assessment via Pancreatic Lipase Inhibition

The process for measuring inhibition activity is detailed in previous works [32,33]. Initially, crude pig or porcine pancreatic lipase (PPL) at a 1 mg/mL concentration was prepared in a 50 mM phosphate buffer solution with a pH of 7.0 and then centrifuged at 12,000× *g* to discard any non-soluble matter. The resulting clear supernatant was then diluted using the same buffer to obtain a final enzyme concentration of 0.1 mg/mL, following protocols established in earlier research [32,33]. To assess inhibition, concentrations of 35, 70, 105, 140, and 175 μg/mL of the samples were placed into a 96-well microplate, and metformin and orlistat, each at a concentration of 18 μM, were used as controls. This was followed by adding 20 μL of 10 mM *p*-nitrophenyl butyrate (pNPB) substrate into each well. The reaction mixture was incubated at 37 °C for 10 min. Orlistat (C_29_H_53_NO_5_, PubChem CID: 3034010), a known inhibitor of PPL, served as a standard for comparison. The activity was assessed by monitoring absorbance at 405 nm using a DR-200Bc ELISA microplate reader, with enzymatic activity defined by the release of 1 mol of *p*-nitrophenol (4-nitrophenol, C_6_H_5_NO_3_) per minute at 37 °C. The reduction in PPL activity in the assay mixture quantified the inhibitory effect on lipase. To ensure the reliability of the data, each test was conducted in triplicate (*n* = 3).

$$Inhibition\;of\;Lipase\;Activity\;\left(\%\right)=100-\frac{B-B_{c}}{A-A_{c}}\times 100\%$$

*A* = activity without inhibitor; *B* = activity with inhibitor; *A_c_* = negative control (−) without inhibitor; and *Bc* = negative control (−) with inhibitor.

#### 2.4.2. Antidiabetic Assessment via α-Glucosidase and α-Amylase Inhibition

Two different inhibitory activity tests were performed on the samples as per methodologies described in previous studies in the literature, and the drug acarbose was used as a positive control in these antidiabetic experiments [32,33]. A 50 mL volume of phosphate buffer solution (pH: 6.9) containing the enzyme α-glucosidase was produced for the α-glucosidase inhibition experiment. The solution’s enzyme concentration was 1.52 UI/mL. Maltose and sucrose solutions were added to the mixture, and then samples were added at various concentrations (ranging from 40 to 200 μg/mL); metformin and acarbose, each at a concentration of 18 μM, were used as controls. After that, each sample was combined and incubated for 20 min at 37 °C. The tubes were then heated to 100 °C for two minutes in order to deactivate the enzyme. In the α-amylase inhibition experiment, 0.5 mg/mL of pig/porcine pancreatic amylase, 0.006 M sodium chloride (NaCl), sodium phosphate buffer (Na_2_HPO_4_; pH 6.9), and diluted FOE samples were incubated at five different concentrations ranging from 40 to 200 μg/mL. After that, each 500 μL mixture of 1% starch solution for was incubated 10 min at room temperature (25 °C). To finish the reaction, 3,5-dinitro salicylic acid (CAS 609-99-4) was added, and the mixture was then incubated for five minutes at 100 °C. Each FOE sample’s absorbance was measured at 540 nm following its dilution with distilled water and cooling to 22 °C.

#### 2.4.3. Cell Culture and Cell Viability of 3T3-L1 Mouse Cells

On the first day, the modification proposed by Zhang et al. and Jeong and Park [34,35] for the culture and differentiation of 3T3-L1 mouse cells was tested by initially seeding 3T3-L1 preadipocyte cells in 6-well plates at a density of 1 × 10^5^ cells per well and then culturing them in DMEM with 10% FBS at 37 °C for 24 h in a 5% carbon dioxide atmosphere. On the second day, the 3T3-L1 cells were treated with a differentiation medium that included 10% FBS in DMEM along with 0.5 mM of 3-isobutyl-1-methylxanthine (IBMX), 1 μM of dexamethasone, and 10 μg/mL of insulin in order to accomplish adipocyte differentiation. It took three days to complete this process. In order to preserve adipocyte features, a fresh insulin medium containing DMEM with 10% FBS and 10 ug/mL of insulin was added on day 5 and incubated for three days. The impact of simvastatin (1.5, 3, and 6 μM (35, 70, 105, 140, and 175 μg/mL)) and FOE (0.25, 0.5, and 1 mM (35, 70, 105, 140, and 175 μg/mL)) was then evaluated using assays conducted in triplicate in a differentiation medium.

To evaluate the vitality of the 3T3-L1 preadipocyte cells (American Type Culture Collection, Manassas, VA, USA), the MTT reduction test was employed. The first step in this process was to culture the cells in a 96-well plate at 37 °C for 24 h in 5% carbon dioxide using Dulbecco’s modified Eagle’s medium (DMEM) with 10% FBS (fetal bovine serum) at a density of 5 × 10^3^ cells per well. This was the environment prior to treatment with various extract concentrations and simvastatin for 72 h of. Following incubation, 100 μL of MTT solution (5 mg/mL) was added to each well, and the mixture was then incubated for an additional four hours at 37 °C. After dissolving MTT–formazan crystals in live cells with 100 μL of DMSO, the absorbance at 540 nm was measured. The data from the treatment wells for viable cells were then compared to the outcomes of the control wells to determine the percentage of cell viability.

##### In Vitro Assessment of MAPK8, PPARG, HMGCR, CPT-1, and GLP1 Expression on Preadipocyte 3T3-L1 Mouse Cells

Following established research experimental guidelines/protocols and the manufacturer’s instructions (Elabscience^®^ Elabscience Biotechnology Co., Ltd., Wuhan, China), an in vitro examination of the expression of MAPK8 or JNK1, PPARG, HMGCR, CHPT1 or CPT1, and GLP1 was performed [36] with modifications. A polyvinylidene difluoride membrane was treated with a blocking solution made of 5% dry skim milk in a Tris-with-Tween (T-TBS) saline buffer in order to identify MAPK8, PPARG, HMGCR, CPT1, and GLP1. This action was taken to stop any detection reagents from being absorbed by the membrane. This buffer has a pH of 7.4, 20 mmol/L of Tris-HCl, 0.138 mol/L of sodium chloride (NaCl; Sigma Aldrich, Darmstadt, Germany), and a concentration of 0.1% Tween 20. In order to identify phosphorylated MAPK8, PPARG, HMGCR, CPT1, and GLP1, the membrane was treated with a blocking solution consisting of 5% albumin (more precisely, bovine serum albumin, or BSA) in T-TBS. This was carried out in order to identify phosphorylated protein. A unique methodology was used to evaluate the expression of MAPK8, PPARG, HMGCR, CPT1, and GLP1. First, primary antibodies were applied to the cell membrane, and then secondary antibodies linked to peroxidase were added. The T-TBS solution was diluted with a solution containing 5% bovine serum albumin (BSA) to dilute the primary and secondary antibodies. This thorough antibody-based method was used to ensure accuracy through antibody dilution and suitable incubation conditions while gaining insight into the expression of MAPK8, PPARG, HMGCR, CPT1, and GLP1. The experimental procedure included planting 5000 3T3-L1 cells into wells, using 100 μL/well, in order to finalize the results. Over the course of a 24 h incubation period, these cells were treated with FOE at concentration of 1 mM. Subsequently, the collected data were examined to determine the percentage value in comparison to the control group (a group of cells that received neither treatment nor 0 mM of FOE). Optical density (OD) measurements at wavelengths of 665 nm and 620 nm, utilizing spectrophotometers (SmartSpec Plus from Bio-Rad Laboratories. Inc., Hercules, CA, USA), facilitated this percentage (%) value assessment.

### 2.5. Data Analytics and Management

The MacBook version of GraphPad Prism Premium 10 (GraphPad Software, Inc., San Diego, CA, USA) was used to perform a statistical analysis of the data. The data distribution is assessed using the Shapiro–Wilk test. To determine the average difference between treatment groups, a one-way ANOVA test was used if the data were normally distributed (significance < 0.05); if they were not, the Kruskal–Wallis test was conducted. Using the statistical analysis package GraphPad Premium, the lethal dose, 50% (also known as the lethal concentration 50 or LC_50_), of lipase, α-glucosidase, and α-amylase due to their inhibitory activities was analyzed using Non-linear regression (log(inhibitor) vs. normalized response–variable slope) and to determine the significance value (95%CI) of miR-21/132 expression through a two-way ANOVA test.

## 3. Results

### 3.1. In Silico Study Results

#### 3.1.1. List of Compounds and Peptides after Metabolomic Profiling

The metabolite profile of *E. bulbosa* was successfully obtained and analyzed using a non-targeted metabolic profile UPLC-ESI-MS/MS analysis (Table 1 for metabolites) and ethanol solvent maceration (Table 2 for peptides). From the FOE, 10 compounds and 11 peptides were obtained and observed. All observed compounds and peptides were then searched for canonical SMILES, which were then used for a protein–protein interaction analysis or a network pharmacology analysis (Table S1).

#### 3.1.2. Pa Score, Toxicity Prediction, Drug-Likeness, and Network Pharmacology Analysis

As previously mentioned, Pa scores, projected toxicity, drug similarity, and network pharmacology analyses were performed on FOE compounds containing the target proteins and metabolic syndrome proteins in order to elucidate the targeting route at the molecular docking stage (Table 3). Based on the data analysis presented in Table 3, there are two compounds and eight peptides that have the potential to become drug candidates targeting metabolic syndrome, including compounds C3 and C6 as well as peptides P1, P2, P3, P4, P5, P6, P8, and P9, which have potential value demonstrated by the Pa value of insulin promoter excretion in relation to metabolic syndrome. This was followed by a predicted LD_50_ > 1000 or a toxicity class > 4, fulfilling Lipinski’s rules, as shown in Table 3.

**Figure 1:**
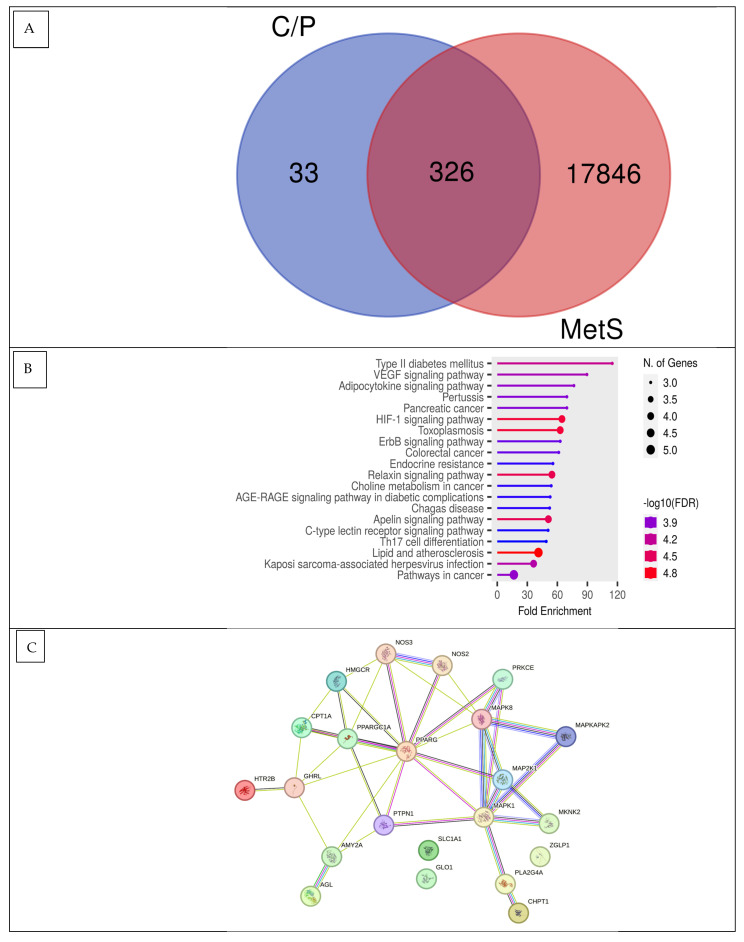
Network pharmacology FOE against metabolic syndrome. (**A**) Venn diagram showing shared FOE targets and genes associated with metabolic syndrome. (**B**) Annotation of gene metabolic biological processes for FOE targets (false discovery rate or FDR < 0.90). (**C**) Protein–protein interactions (PPIs) of FOE targets in metabolic syndrome.

A network pharmacology analysis was carried out to find central receptors that play a role in signaling metabolic syndrome, especially diabetes mellitus. In the disease-related analysis of the acquisition and targets of FOE mapped on the Venn diagram in Figure 1A, it can be seen that the appropriate intersection targets of FOE and metabolic syndrome are 326 genes and proteins. A further analysis of interactions between the target proteins obtained from the FOE and their relationship with metabolic syndrome yielded several possible signals in the processing of metabolic syndrome, such as metabolic pathways, lipids, atherosclerosis, and regulatory inflammatory mediators (Figure 1B,C), with fold enrichment reaching 4.8 for the lipid and atherosclerosis pathway.

In Table 4, MAPK8, PPARG, and HMGCR were identified as candidate target receptors for FOE and show potential to interact as insulin promoters. It was noted that CPT-1 and GLP-1 were also associated with MAPK8; this implies that FOE is also involved in the metabolic syndrome signaling pathway. Several signaling pathways were also observed that allow for further study. Based on these PPI results, MAPK8, PPARG, HMGCR, CPT-1, and GLP-1 were selected for further testing using molecular docking simulation.

#### 3.1.3. Docking Potency of Compound Found in *Eleutherine bulbosa*

The drug target molecular docking simulation used is shown in (Table 5). The potential compounds and peptides of FOE identified were used as materials for molecular docking with MAPK8, PPARG, HMGCR, CPT-1, and GLP-1 receptors as drug targets, as shown in (Table 5). Metformin, as an anti-metabolic syndrome agent and diabetes mellitus drug, is used as a control compound; its affinity values are shown in (Table 5). All compounds (C3 and C6) and peptides (P1, P2, P3, P4, P5, P6, P8, and P9) demonstrated good control affinity values (better than the control affinity value of metformin as a threshold) for these five receptors.

The performance of the substances contained in FOE against MAPK8, PPARG, HMGCR, CPT-1, and GLP1 can be determined by the binding activity of substances that block signal binding to the receptor, as shown in the Supplementary Materials, Table S2. The performance of these substances can be explained by the strength and number of amino acids they bind. This prevents the signal from binding to the receptor. The number of bound amino acids can explain the flexibility of use of a material, and the strength of various chemical bonds, namely hydrogen bonds, can explain the affinity of a material. Most of the substances contained in FOE are tuber compounds (C3 and C6) and peptides (P1, P2, P3, P4, P5, P6, P8, and P9) both of which express hydrogen bonds with amino acids that play a role in the MAPK8, PPARG, HMGCR, CPT-1, and GLP-1 signaling pathways. This explains the differences in the level of docking activity of each substance based on its form and chemical activity.

### 3.2. In Vitro Study Results

#### 3.2.1. Lipase Inhibition Potential of FOE

**Figure 2:**
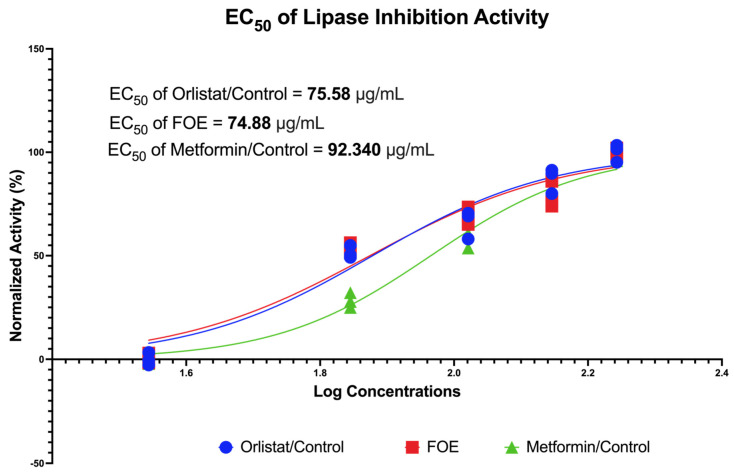
Dose-dependent inhibition of pancreatic lipase activity of forest onion extract, orlistat, and metformin. Inhibitory activity was measured at concentrations of 35, 70, 105, 140, and 175 μg/mL.

The inhibitory activities of FOE against porcine pancreatic lipase are shown in Figure 2. The FOE inhibited pancreatic lipase activity at concentrations of 35, 70, 105, 140, and 175 μg/mL, respectively. In this study, the controls used were orlistat and metformin, which act as anti-obesity agents. The FOE, orlistat, and metformin had $IC_{50}$ values of 74.88 μg/mL, 75.58 μg/mL, and 92.34 μg/mL, respectively. This indicates that the FOE could inhibit pancreatic lipase activity with higher inhibitory efficacy, even at a lower concentration, than the control.

#### 3.2.2. α-Glucosidase and α-Amylase Potential of FOE

**Figure 3:**
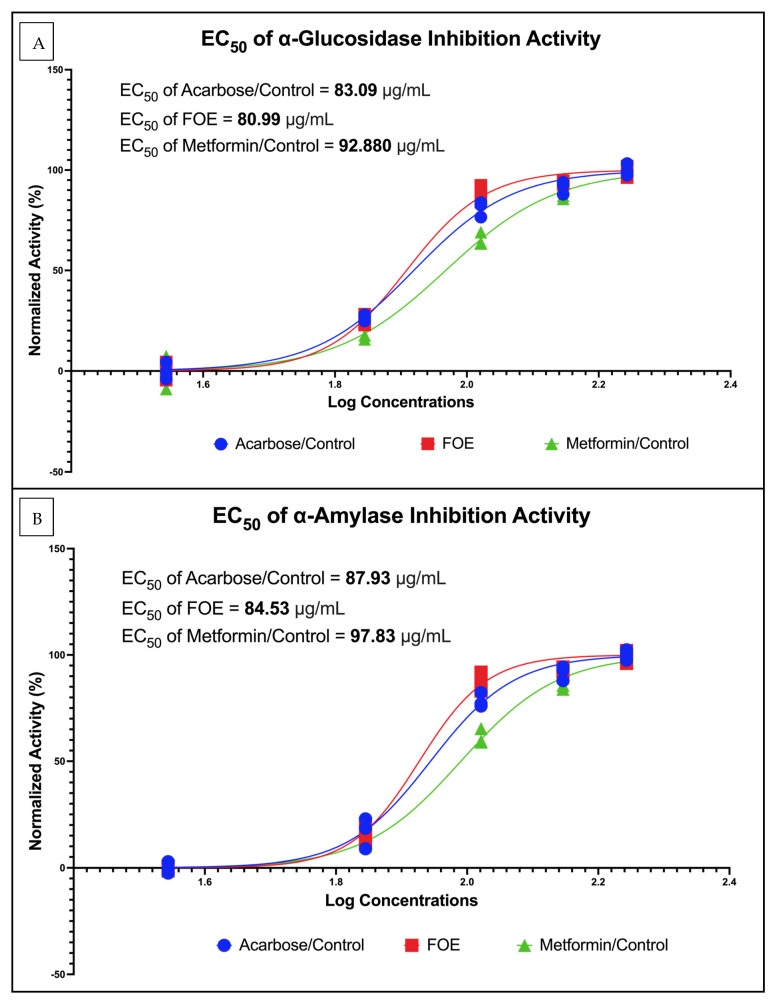
(**A**) Inhibitory activities of FOE against α-glucosidase and (**B**) α-amylase. Dose-dependent inhibition of α-glucosidase and α-amylase activity of forest onion extract, acarbose, and metformin. Inhibitory activity was measured at concentrations of 35, 70, 105, 140, and 175 μg/mL.

The inhibitory activities of the FOE against α-glucosidase and α-amylase are shown in Figure 3. The FOE inhibited α-glucosidase activity at concentrations of 35, 70, 105, 140, and 175 μg/mL. In this study, the controls used were acarbose and metformin, which act as antidiabetic agents. The FOE, acarbose, and metformin had $IC_{50}$ values of 80.99 μg/mL, 83.09 μg/mL, and 92.88 μg/mL, respectively but the FOE inhibited α-amylase activity at the same concentrations as α-glucosidase. The FOE, acarbose, and metformin had $IC_{50}$ values of 84.53 μg/mL, 87.93 μg/mL, and 97.83 μg/mL, respectively. This indicates that the FOE was able to inhibit α-glucosidase and α-amylase activity with higher inhibitory efficacy, even at a lower concentration than the control.

#### 3.2.3. Downregulation of Protein Expression and Reduction in 3T3-L1 Mouse Cells by FOE

**Figure 4:**
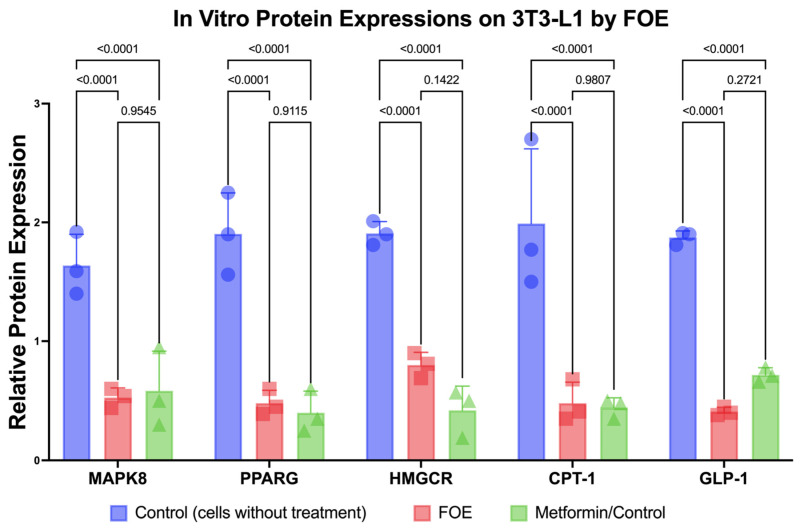
Inhibition of protein expression (MAPK8, PPARG, HMGCR, CPT-1, and GLP-1) by forest onion extract, untreated cells, and metformin. Values represent mean ± SD values of triplicate measurements.

Reductions in the protein expression levels of MAPK8, PPARG, HMGCR, CPT-1, and GLP1 due to the FOE are shown in Figure 4. The FOE generally inhibited protein expression. In this study, control cells without treatment and with metformin were used. The FOE was able to reduce the protein expression of MAPK8, PPARG, HMGCR, CPT-1, and GLP1 compared to untreated cells. While the FOE reduced the expression of the HMGCR protein, it was not as effective as metformin. However, the protein expression levels of MAPK8, PPARG, and CPT-1 showed relatively similar results to metformin. The FOE showed superior results in reducing GLP-1 protein expression compared to both untreated cells and those treated with metformin. This indicates that the FOE was able to decrease the protein expression levels of MAPK8, PPARG, HMGCR, CPT-1, and GLP1, even at lower concentrations than the control.

**Figure 5:**
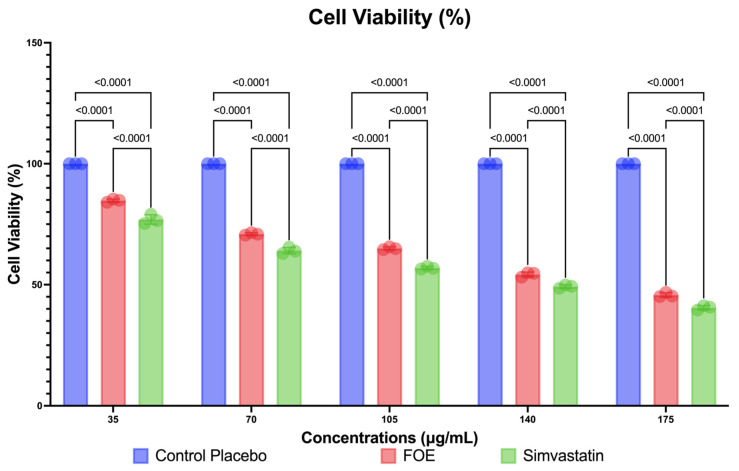
Reduction in viable cells (3T3-L1) by FOE, placebo, and simvastatin. Activity was measured at concentrations of 35, 70, 105, 140, and 175 μg/mL.

The FOE can also reduce the number of viable 3T3-L1 cells, as shown in Figure 5. The FOE reduced the viable cells at concentrations of 35, 70, 105, 140, and 175 μg/mL, respectively. In this study, control cells were treated with simvastatin. The FOE reduced viable cells more effectively than the placebo, but the decreasing level of viable cells showed similar results to simvastatin. While concentrations of the FOE of 105 μg/mL and greater have a detrimental effect on cell viability (close to 50%), these results are still lower than those achieved with simvastatin, a generally available medicine for lowering cholesterol. This indicates that the FOE was able to decrease viable 3T3-L1 cells, even at lower concentrations than the control.

## 4. Discussion

**Figure 6:**
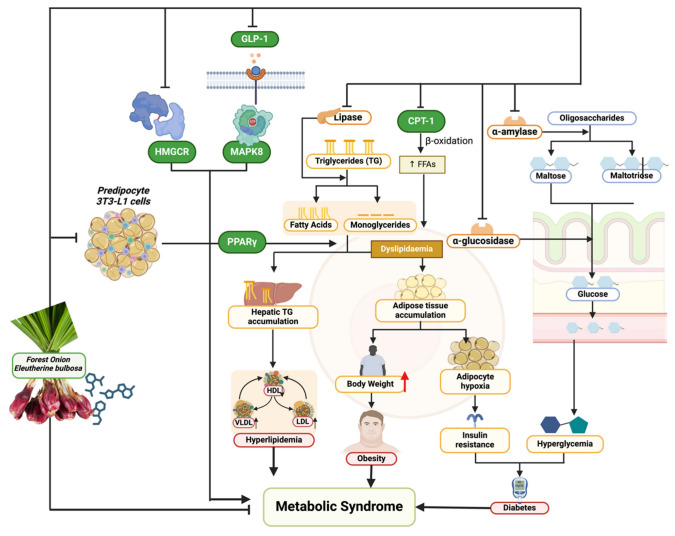
Biomechanism of FOE in combating metabolic syndrome. Created by Fahrul Nurkolis using BioRender.com Premium License.

This study delves into a natural product’s pharmacological properties, focusing on the complex and widespread problem of metabolic syndrome. This study’s novel method combines computational predictions with empirical in vitro validation to fill a crucial gap in the search for comprehensive and side-effect-free treatments. Forest onion, scientifically known as *E. bulbosa*, have been underappreciated as a functional food despite their well-documented antioxidant and anticancer characteristics [37]. This study’s exploration of their possible anti-metabolic syndrome effect is timely and important (Figure 6).

Metabolic syndrome’s pathophysiology is characterized by an intricate interplay of metabolic pathways such as insulin signaling, lipid metabolism, glucose regulation, and inflammation [38]. Disruption to these networks leads to the characteristic traits of metabolic syndrome: high blood glucose, high blood pressure, abnormal lipid levels, and obesity [38,39]. This study discovered that FOE inhibits important enzymes like pancreatic lipase, α-glucosidase, and α-amylase and influences the expression of proteins such as MAPK8, PPARG, HMGCR, CPT-1, and GLP1. This highlights the diverse ways in which these phytochemicals address metabolic syndrome. Inhibiting pancreatic lipase directly affects lipid metabolism by limiting the breakdown of dietary fats, leading to a decrease in the absorption of triglycerides, which is crucial in treating obesity and dyslipidemia [39]. Inhibiting α-glucosidase and α-amylase enzymes delays carbohydrate digestion, resulting in lower postprandial glucose levels, which directly impacts glucose regulation and helps control hyperglycemia [40]. Moreover, the decrease in proteins like MAPK8 indicates a weakening of the stress-activated pathways that play a role in insulin resistance, a fundamental aspect of metabolic syndrome [41]. The nuclear receptor PPARG is crucial in regulating lipid metabolism and glucose balance [42]. Regulating PPARG expression may impact adipocyte development and fatty acid storage, thereby treating obesity on a cellular scale [42]. HMGCR is a key enzyme in the production of cholesterol. Its inhibition helps treat dyslipidemia by decreasing the production of cholesterol in the body [43]. CPT-1, which plays a role in the mitochondrial breakdown of long-chain fatty acids, is a potential target for regulating fatty acid metabolism and understanding obesity and energy balance [44]. GLP1 (glucagon-like peptide-1) affects insulin secretion, appetite, and stomach emptying, suggesting that controlling GLP1 could significantly impact glucose control and weight management [45,46].

Molecular docking simulations showed that FOE has strong affinities for these proteins, indicating that these phytochemicals can interact with and influence the function of these important enzymes and receptors in relation to metabolic syndrome pathogenesis. This is especially convincing in the context of how natural substances influence the signaling networks that control metabolic activities. These chemicals attach to target proteins and can trigger a series of molecular interactions which can activate or inhibit downstream pathways that correct the metabolic abnormalities associated with metabolic syndrome [46].

These findings are inherently connected to the distinct metabolic composition of the forest onion. Flavonoids, recognized for their antioxidant effects, have been linked to enhancing insulin sensitivity and decreasing inflammation, which are crucial elements in the development of metabolic syndrome [47]. The flavonoids in FOE may impact the proteins MAPK8 and PPARG, which are related to inflammatory responses and lipid metabolism.

Anthocyanins, a category of chemicals present in forest onions, have been linked to anti-obesity and antidiabetic properties in many studies [48]. The effects may be influenced by processes such as increased lipid metabolism and higher glucose absorption, which correspond with the reported inhibitory effects on pancreatic lipase and α-glucosidase [48,49]. FOE may modulate HMGCR expression through anthocyanins, which are known for their ability to regulate cholesterol levels [48,49].

Quinones enhance the therapeutic potential of *E. bulbosa* against metabolic syndrome due to their strong antioxidant and anti-inflammatory properties [49]. The ability of FOE to decrease the expression of important proteins such as CPT-1 and boost GLP1 activity explains its antidiabetic and lipid-lowering benefits [44,48,49].

This study’s results emphasize that the biochemical features of FOE can support a comprehensive method of addressing metabolic syndrome. The intricate relationship between the plant’s chemicals and the biological targets associated with metabolic syndrome explains how natural substances can provide a multifaceted treatment approach. FOE targets lipid metabolism, glucose homeostasis, and inflammatory processes together to address both the symptoms and the underlying causes of metabolic syndrome.

The strength of this study lies in its ability to illuminate the specific biomolecular interactions that underpin the therapeutic potential of *E. bulbosa*, providing a blueprint for the development of targeted therapies that address the root causes of metabolic syndrome. However, it is crucial to acknowledge the limitations inherent in extrapolating in vitro findings to in vivo contexts. The cellular environment is complex, and the efficacy observed in vitro may not directly translate to clinical effectiveness.

Future studies should clarify these biomolecular processes in living organisms, utilizing animal models and clinical studies, to confirm the therapeutic benefits of *E. bulbosa* phytochemicals. These investigations will help connect laboratory findings with practical medical uses, leading to the creation of new treatments that utilize complex molecular pathways influenced by natural substances to combat metabolic syndrome.

The study successfully links the distinct phytochemical composition of *E. bulbosa* with its ability to influence important biomolecular pathways in metabolic syndrome. This study emphasizes the importance of forest onions in the search for natural treatments and paves the way for more research into their potential to address complicated metabolic problems.

## 5. Conclusions

This study provides comprehensive insights into the potential of forest onion extract (FOE) to modulate lipid and glucose metabolism both in silico and in vitro. Through a combination of computational analysis, molecular docking, and experimental validation using 3T3-L1 mouse cells, we uncovered a spectrum of bioactive compounds within FOE, shedding light on its therapeutic potential. Notably, our findings demonstrate that FOE exerts inhibitory effects on key enzymes involved in lipid and glucose metabolism, including pancreatic lipase, α-glucosidase, and α-amylase, as evidenced by in vitro assays. Moreover, utilizing preadipocyte 3T3-L1 mouse cells, we elucidated the inhibition of protein expression related to critical metabolic pathways, including MAPK8, PPARG, HMGCR, CPT-1, and GLP-1.

These results underscore the promising role of FOE as a functional food targeting metabolic syndrome. Moving forward, further investigations are warranted to validate these findings in vivo and in human clinical trials, utilizing doses consistent with those observed in this study. Such endeavors hold potential for elucidating the therapeutic efficacy of FOE and its metabolites in managing metabolic disorders and improving overall metabolic health.

## 6. Patents

The extraction method resulting from the work reported in this article has been registered as a patent by Fahrul Nurkolis in Indonesia.

## Figures and Tables

**Table 1:** Metabolite compounds observed in FOE.

No.	Observed Compounds	Molecular Formula	Structural Class	RT (Min)	Observed MW (*m*/*z*)	PubChem ID or Substance ID
C1	*p*-Cresol	C_7_H_8_O	Aromatic Alcohol	6.993	108.05783	2879
C2	4-Fluorophenol	C_6_H_5_FO	Aromatic Halide	0.678	112.03258	9732
C3	Guvacine	C_6_H_9_NO_2_	Amino Acid	0.832	127.06334	3532
C4	Furaneol	C_6_H_8_O_3_	Ester	0.854	128.04738	19309
C5	*p*-cymene	C_10_H_14_	Bicyclic Hydrocarbon	5.345	134.10948	7463
C6	Elaeokanine C	C_12_H_21_NO_2_	Amine	11.249	211.15705	442855
C7	Flavokawain A	C_18_H_18_O_5_	Anthraquinone	7.357	314.11498	5355469
C8	3-Hydroxy-3,4-bis[(4-hydroxy-3-methoxyphenyl)methyl]oxolan-2-one	C_20_H_22_O_7_	Polyphenol	4.171	374.13597	321311
C9	Euparin	C_13_H_12_O_3_	Phenolic Acid	6.218	216.0783	119039
C10	Eleutherol	C_14_H_12_O_4_	Phenolic Aldehyde	8.492	244.07336	120697

**Table 2:** Peptides observed in FOE.

Type	Observed Peptides	Molecular Formula	RT (Min)	Observed MW (*m*/*z*)	PubChem ID or Substance ID
P1	Gly-Leu	C_8_H_16_N_2_O_3_	0.872	188.11589	92843
P2	Ala-Leu	C_9_H_18_N_2_O_3_	2.847	202.13164	96801
P3	Val-Ser	C_8_H_16_N_2_O_4_	0.874	204.11071	139506
P4	Gly-Phe	C_11_H_14_N_2_O_3_	3.223	222.10013	92953
P5	Ala-phe	C_12_H_16_N_2_O_3_	2.809	236.11589	96814
P6	Asp-Leu	C_10_H_18_N_2_O_5_	2.946	246.1213	332962
P7	Val-Met	C_10_H_20_N_2_O_3_S	2.62	248.1192	292427
P8	Ala-Tyr	C_12_H_16_N_2_O_4_	1.948	252.11066	92946
P9	Lys-Leu	C_12_H_25_N_3_O_3_	1.391	259.18932	7016103
P10	Arg-Leu	C_12_H_25_N_5_O_3_	1.508	287.19524	6992563
P11	Arg-Glu	C_11_H_21_N_5_O_5_	0.804	303.15392	6995004

**Table 3:** The evaluation of FOE potential for anti-metabolic syndrome based on structure–activity relationship (SAR) predictions, Pa Score, Toxicity Prediction, Drug-Likeness, and Network Pharmacology Analysis.

Compounds/Peptides	Pa Score	Toxicity Model Computation Analysis		Drug-Likeness		
	Insulin Promoter	Predicted LD_50_ (mg/kg)	Toxicity Class	Lipinski Rule	Pfizer Rule	GSK
C1	0.605	160	3	Accepted	Accepted	Accepted
C2	0.433	270	3	Accepted	Accepted	Accepted
C3	0.457	1000	4	Accepted	Accepted	Accepted
C5	0.751	3	1	Accepted	Rejected	Accepted
C6	0.559	338	4	Accepted	Accepted	Accepted
P1	0.563	6838	6	Accepted	Accepted	Accepted
P2	0.608	5000	5	Accepted	Accepted	Accepted
P3	0.659	5000	5	Accepted	Accepted	Accepted
P4	0.678	1000	4	Accepted	Accepted	Accepted
P5	0.724	1000	4	Accepted	Accepted	Accepted
P6	0.634	6836	6	Accepted	Accepted	Accepted
P8	0.596	1000	4	Accepted	Accepted	Accepted
P9	0.405	5000	5	Accepted	Accepted	Accepted

**Table 4:** Results of the top one protein–protein interaction (PPI) network analyses.

Name	Degree	Betweenness Centrality	Closeness Centrality	Overall Score	Pathway
PPARG	10	0.32564103	0.8125	11.138141	Peroxisomal beta-oxidation pathway of fatty acids; tissue-specific adipocyte P2 (aP2) enhancer
MAPK8	7	0.25769231	0.68421053	7.94190283	Protein kinase/c-Jun *N*-terminal kinase (SAP/JNK) signaling pathway; MAP2K4/MKK4 and MAP2K7/MKK7 phosphorylate and activate MAPK8/JNK
HMGCR	4	0.01495726	0.54166667	4.55662393	Cholesterol biosynthesis; obesity
GLP-1	4	0.01068376	0.54166667	4.55235043	Adenylyl cyclase is activated and intracellular cAMP levels are raised as a result of ligand binding activating a signaling cascade; diabetes and insulin

**Table 5:** ΔG of molecular docking parameters of identified compounds/peptides from FOE.

Compounds/Peptides and Control as Ligands	MAPK8 (3ELJ)	PPARG (8BF1)	HMGCR (2R4F)	CPT-1 (1NDB)	GLP-1 (4ZGM)
Control Metformin (4901)	−4.5	−4.9	−5.6	−5.4	−4.5
Control Orlistat (3034010)		−6.6	−6.4		
Control Simvastatin (54454)			−7.7		
C3	−4.9	−5.1	−6.0	−5.1	−4.6
C6	−6.0	−6.4	−6.1	−6.2	−5.3
P1	−5.4	−5.3	−6.0	−6.5	−5.1
P2	−5.4	−5.8	−6.4	−6.5	−5.2
P3	−5.6	−5.4	−5.9	−6.1	−4.8
P4	−7.5	−6.3	−6.6	−7.9	−6.1
P5	−7.0	−6.3	−6.9	−7.9	−6.2
P6	−6.3	−6.0	−6.1	−6.7	−5.8
P8	−7.1	−6.8	−7.2	−7.8	−6.2
P9	−5.5	−5.5	−5.8	−6.6	−5.1

## Data Availability

The datasets presented in this study can be requested from the corresponding author, F.N., or R.A.S. due to legal and ethical reasons.

## Supplementary Materials

The following supporting information can be downloaded at https://www.mdpi.com/article/10.3390/nu16101441/s1. Table S1: SMILES canonical for EBE metabolites and peptides; Table S2: Full visualization of amino acid interaction of FOE properties against MAPK8, PPARG, HMGCR, CPT-1, and GLP-1.
