# Supplementary material for: Novel Functional Food Properties of Forest Onion (Eleutherine bulbosa Merr.) Phytochemicals for Treating Metabolic Syndrome: New Insights from a Combined Computational and In Vitro Approach
Source: Nutrients. 2024 May 10;16(10):1441. doi: 10.3390/nu16101441 (PMC11124081; doi:10.3390/nu16101441)
Supplement: Supplementary file 1 [file nutrients-16-01441-s001.zip › nutrients-2929885-supplementary.pdf]

Tabel S1. Compounds and Peptides SMILE Canonical from FOE

Compounds	**Compounds**	**SMILES Canonical**
C1	p-Cresol	CC1=CC=C(C=C1)O
C2	4-Fluorophenol	C1=CC(=CC=C1O)F
C3	Guvacine	C1CNCC(=C1)C(=O)O
C4	Furaneol	CC1C(=O)C(=C(O1)C)O
C5	p-cymene	CC1=CC=C(C=C1)C(C)C
C6	Elaeokanine C	CCCC(=O)C1C2CCCN2CCC1O
C7	Flavokawain A	COC1=CC=C(C=C1)C=CC(=O)C2=C(C=C(C=C2OC)OC)O
C8	3-hydroxy-3,4-bis[(4-hydroxy-3-methoxyphenyl)methyl]oxolan-2-one	COC1=C(C=CC(=C1)CC2COC(=O)C2(CC3=CC(=C(C=C3)O)OC)O)O
C9	Euparin	CC(=C)C1=CC2=CC(=C(C=C2O1)O)C(=O)C
C10	Eleutherol	CC1C2=C(C=C3C=CC=C(C3=C2O)OC)C(=O)O1

Peptides No	**Peptides**	**SMILES Canonical**
P1	Gly-Leu	CC(C)CC(C(=O)O)NC(=O)CN
P2	Ala-Leu	CC(C)CC(C(=O)O)NC(=O)C(C)N
P3	Val-Ser	CC(C)C(C(=O)NC(CO)C(=O)O)N
P4	Gly-Phe	C1=CC=C(C=C1)CC(C(=O)O)NC(=O)CN
P5	Ala-Phe	CC(C(=O)NC(CC1=CC=CC=C1)C(=O)O)N
P6	Asp-leu	CC(C)CC(C(=O)O)NC(=O)C(CC(=O)O)N
P7	Val-Met	CC(C)C(C(=O)NC(CCSC)C(=O)O)N
P8	Ala-Tyr	CC(C(=O)NC(CC1=CC=C(C=C1)O)C(=O)O)N
P9	Lys-Leu	CC(C)CC(C(=O)O)NC(=O)C(CCCCN)N
P10	Arg-Leu	CC(C)CC(C(=O)O)NC(=O)C(CCCN=C(N)N)N
P11	Arg-Glu	C(CC(C(=O)NC(CCC(=O)O)C(=O)O)N)CN=C(N)N

Table S2. Full Visualization of Amino Acid Interaction of FOE Properties against MAPK8, PPARG, HMGCR, CPT-1, and GLP-1

**Compounds/Peptides and Control as Ligands**	**MAPK8 (3ELJ)**	**PPARG (8BF1)**	**HMGCR (2R4F)**	**CPT-1 (1NDB)**	**GLP-1 (4ZGM)**
Control Metformin (4901)	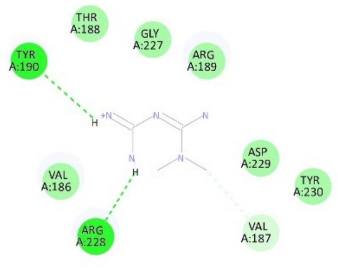	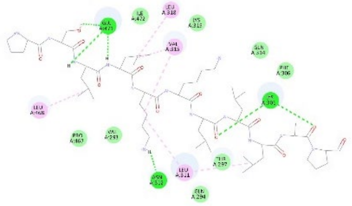	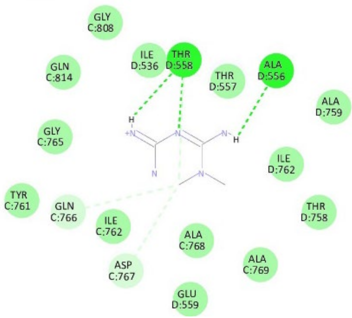	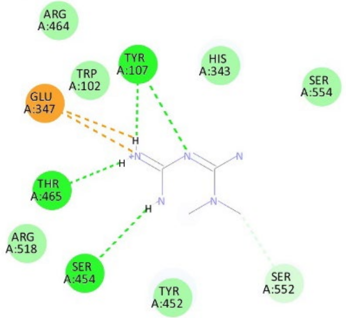	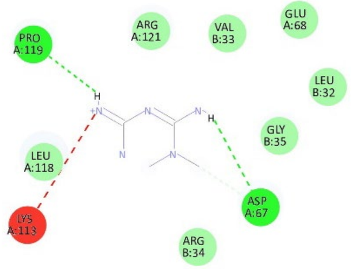
Control Orlistat (3034010)		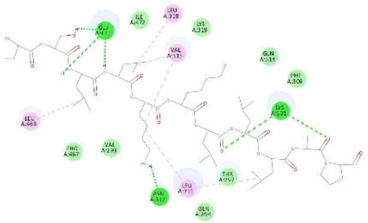	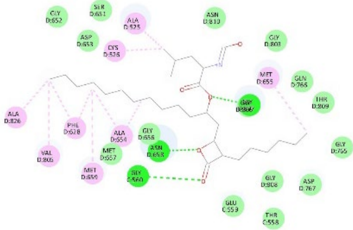		

Control Simvastatin (54454)			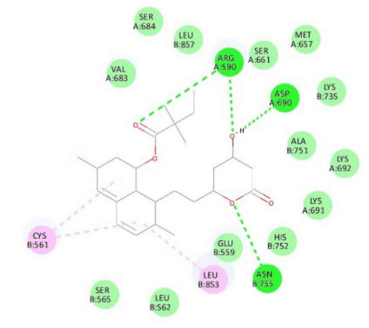		
C3 (3532)	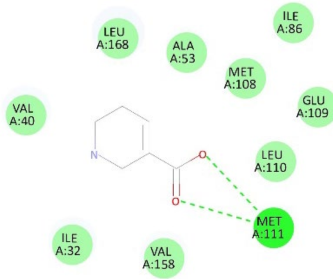	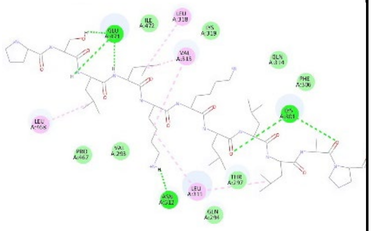	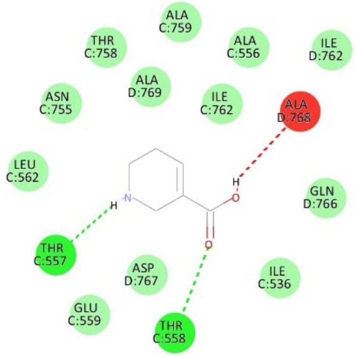	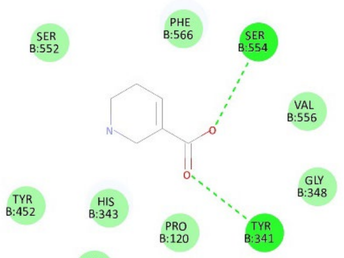	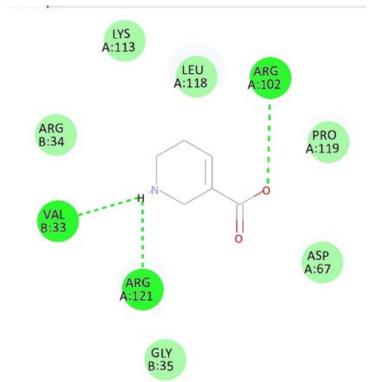

C6 (442855)	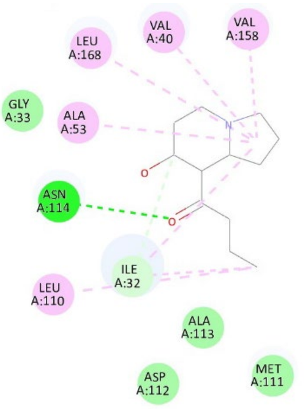	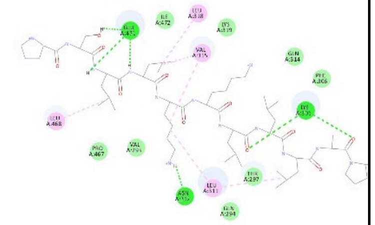	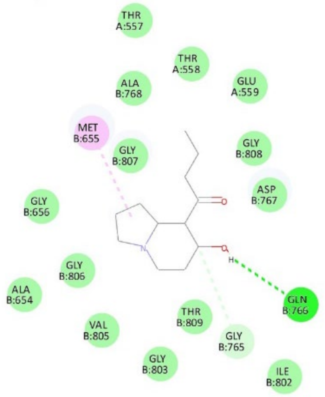	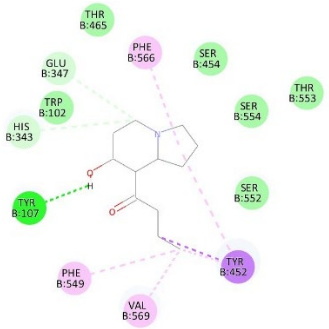	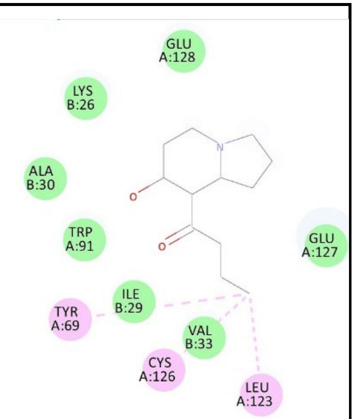
P1	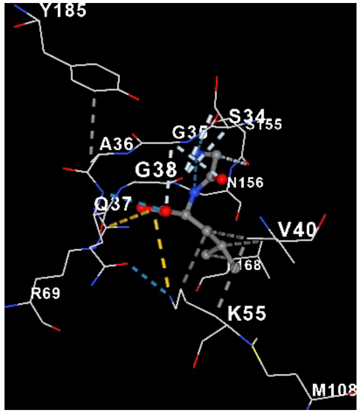	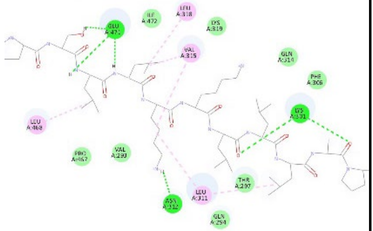	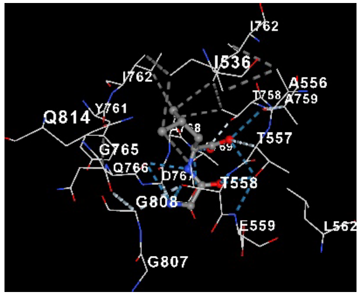	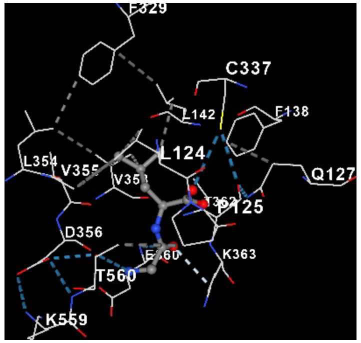	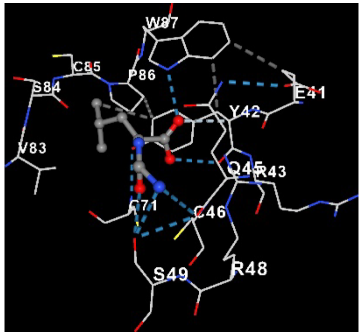

P2	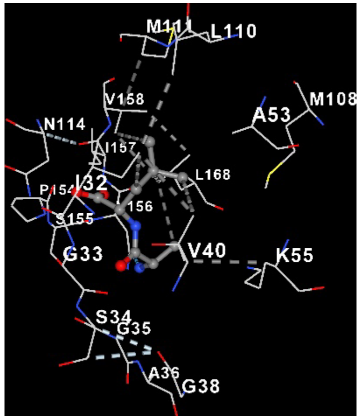M111<br>L110<br>M108<br>A53<br>V158<br>N114<br>I157<br>L168<br>I32<br>P154<br>S155<br>156<br>G33<br>V40<br>K55<br>S34<br>G35<br>A36<br>G38	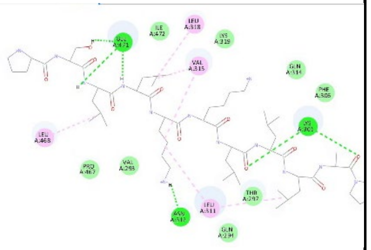	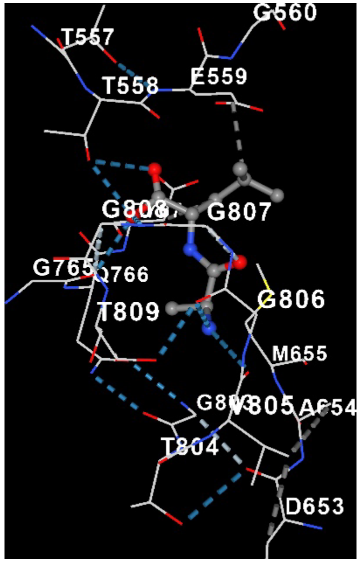G560<br>T557<br>T558<br>E559<br>G808<br>G807<br>G765<br>Q766<br>T809<br>G806<br>M655<br>T804<br>D653	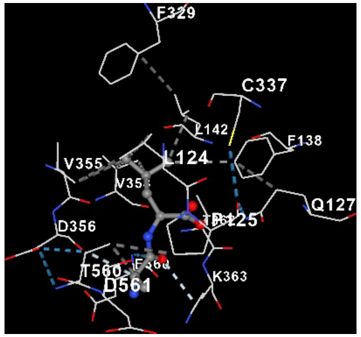F329<br>C337<br>L142<br>F138<br>V355<br>L124<br>V35<br>Q127<br>D356<br>T560<br>D561<br>K363	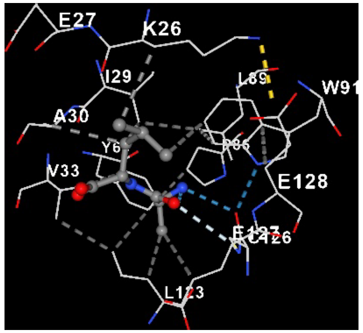E27<br>K26<br>I29<br>L89<br>W91<br>A30<br>Y6<br>V33<br>E128

P3	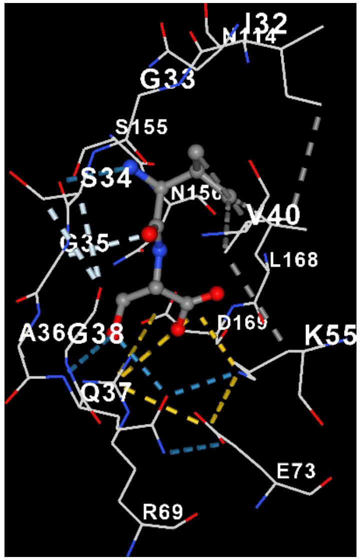 I32 N114 G33 S155 S34 V40 G35 L168 A36 G38 D169 K55 Q37 E73 R69	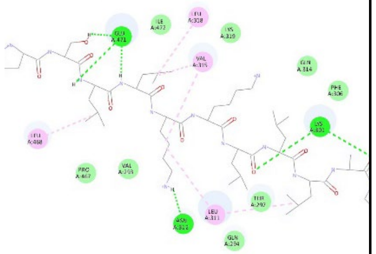	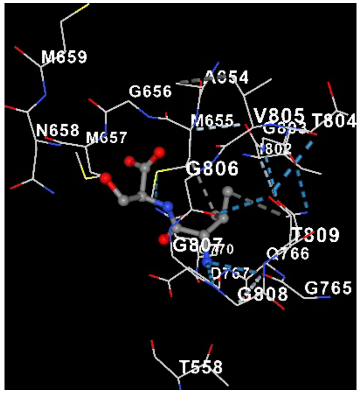 M659 G656 A654 N658 M657 M655 V805 T804 G806 I802 T809 G807 Q766 D767 G765 G808 T558	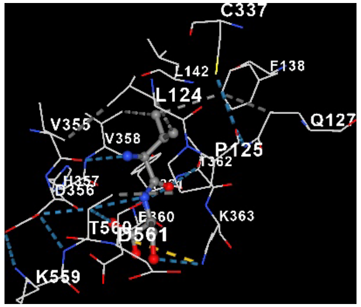 C337 L142 E138 L124 V355 V358 Q127 P125 T362 D356 K363 T560 D561 K559	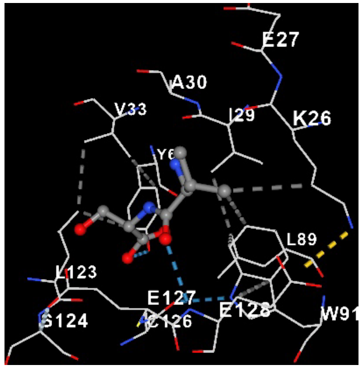 E27 A30 V33 I29 K26 L89 L123 E127 E128 S124 W91
P4	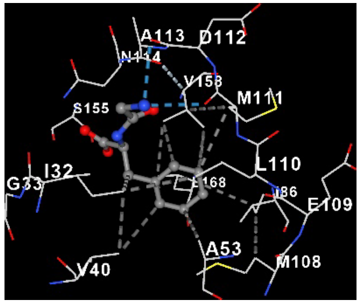 A113 D112 N114 V158 S155 M111 I32 G33 L110 L168 I86 E109 A53 V40 M108	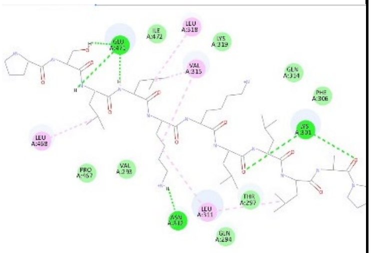	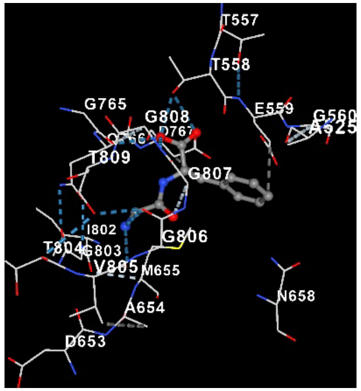 T557 T558 G765 G808 E559 D767 T809 G807 I802 T804 G806 G803 V805 M655 A654 N658 D653	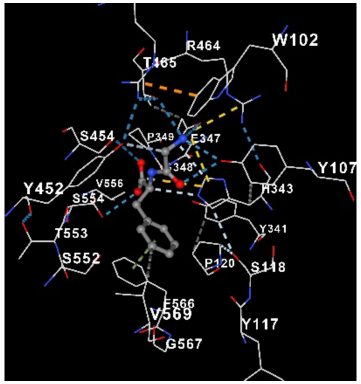 R464 W102 T465 S454 P349 E347 Y107 Y452 V556 S554 H343 T553 Y341 S552 P120 S118 E566 V569 Y117 G567	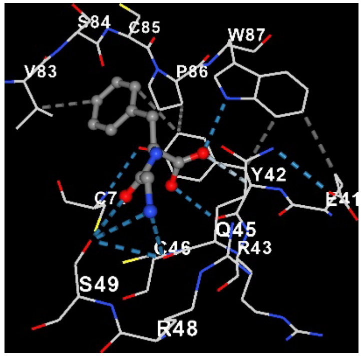 S84 C85 W87 V83 P86 Y42 C7 E41 Q45 C46 R43 S49 R48

P5	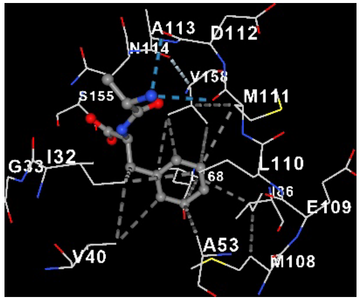A113<br>D112<br>N114<br>V158<br>S155<br>M111<br>G33<br>I32<br>L110<br>E109<br>A53<br>V40<br>M108	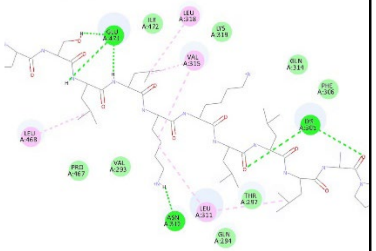	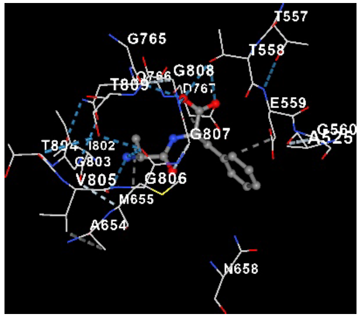T557<br>G765<br>T558<br>G808<br>T809<br>D767<br>E559<br>G807<br>I802<br>G803<br>V805<br>G806<br>M655<br>A654<br>N658	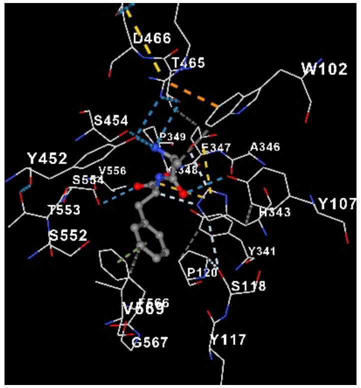D466<br>T465<br>W102<br>S454<br>E347<br>A346<br>Y452<br>V556<br>T553<br>S552<br>H343<br>Y107<br>Y341<br>P120<br>S118<br>G567<br>Y117	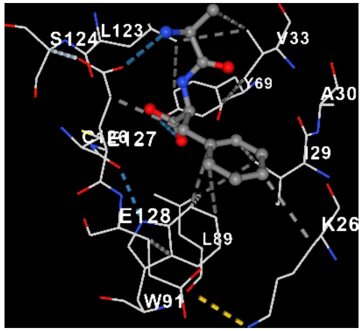S124<br>L123<br>V33<br>Y69<br>A30<br>I29<br>E128<br>L89<br>K26<br>W91
P6	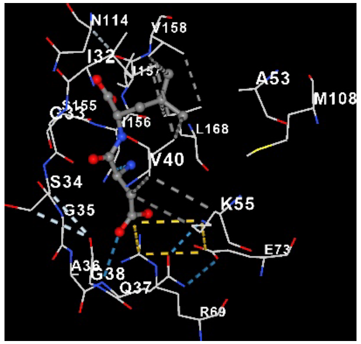N114<br>V158<br>I32<br>A53<br>M108<br>I156<br>L168<br>V40<br>S34<br>G35<br>K55<br>E73<br>G38<br>Q37<br>R69	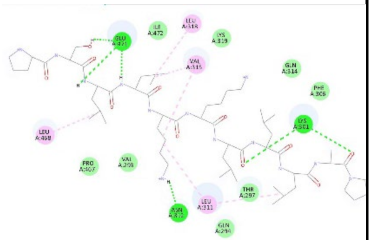	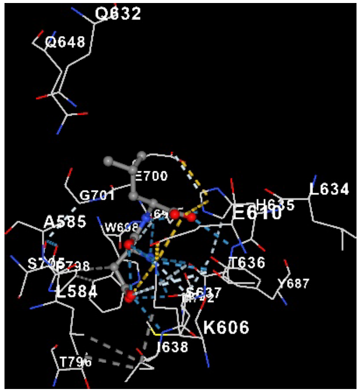Q632<br>Q648<br>E700<br>G701<br>L634<br>A585<br>L584<br>T636<br>K606<br>T796	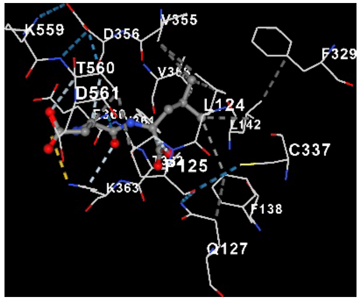K559<br>D356<br>V355<br>F329<br>T560<br>D561<br>L124<br>L142<br>C337<br>P125<br>K363<br>F138<br>Q127	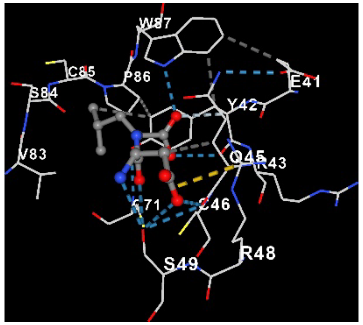W87<br>C85<br>P86<br>S84<br>E41<br>Y42<br>V83<br>Q45<br>R43<br>R48<br>S49

P8	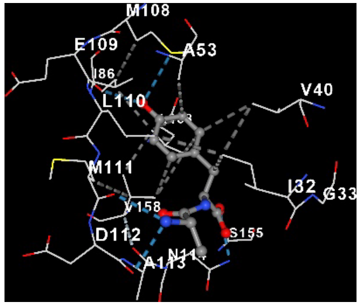	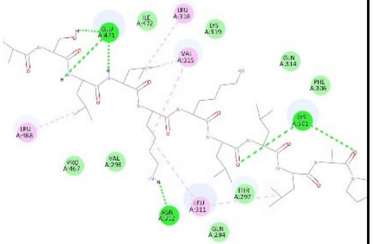	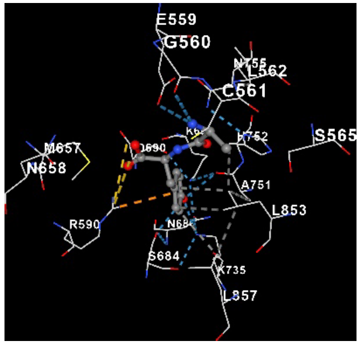	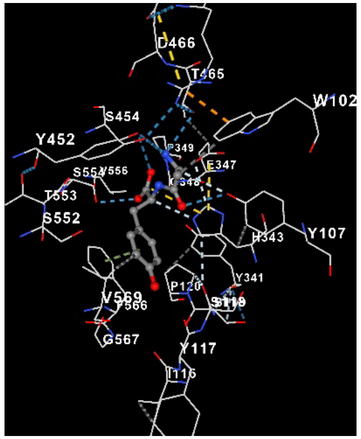	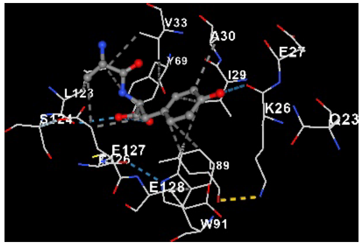
P9	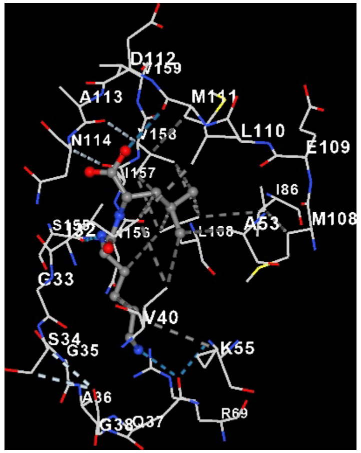	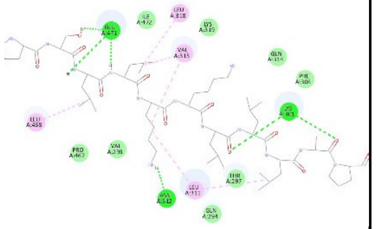	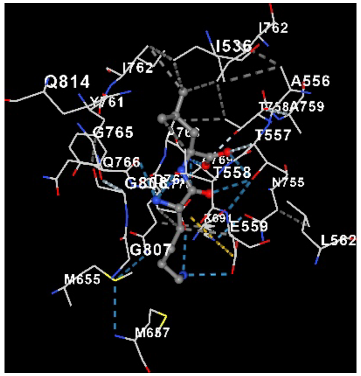	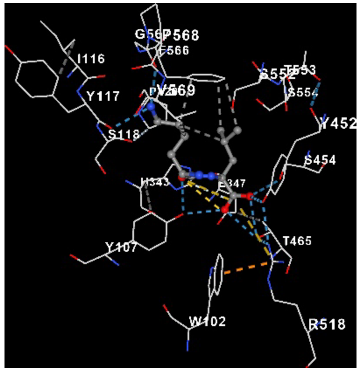	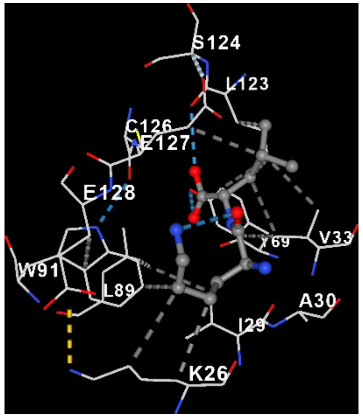
